# Multimodal Fingerprints of Resting State Networks as assessed by Simultaneous Trimodal MR-PET-EEG Imaging

**DOI:** 10.1038/s41598-017-05484-w

**Published:** 2017-07-25

**Authors:** N. J. Shah, J. Arrubla, R. Rajkumar, E. Farrher, J. Mauler, E. Rota Kops, L. Tellmann, J. Scheins, F. Boers, J. Dammers, P. Sripad, C. Lerche, K. J. Langen, H. Herzog, I. Neuner

**Affiliations:** 1 Institute of Neuroscience and Medicine 4, INM-4, Forschungszentrum Jülich, Germany; 2 Institute of Neuroscience and Medicine 11, INM-11, Forschungszentrum Jülich, Germany; 3 Department of Psychiatry, Psychotherapy and Psychosomatics, RWTH Aachen University, Aachen, Germany; 4 JARA – BRAIN – Translational Medicine, Aachen, Germany; 5 Department of Nuclear Medicine, RWTH Aachen University, Aachen, Germany; 6 Department of Neurology, RWTH Aachen University, Aachen, Germany; 7 Monash Institute of Medical Engineering, Department of Electrical and Computer Systems Engineering, and Monash Biomedical Imaging, School of Psychological Sciences, Monash University, Melbourne Victoria, Australia

## Abstract

Simultaneous MR-PET-EEG (magnetic resonance imaging - positron emission tomography – electroencephalography), a new tool for the investigation of neuronal networks in the human brain, is presented here for the first time. It enables the assessment of molecular metabolic information with high spatial and temporal resolution in a given brain simultaneously. Here, we characterize the brain’s default mode network (DMN) in healthy male subjects using multimodal fingerprinting by quantifying energy metabolism via 2- [^18^F]fluoro-2-desoxy-D-glucose PET (FDG-PET), the inhibition – excitation balance of neuronal activation via magnetic resonance spectroscopy (MRS), its functional connectivity via fMRI and its electrophysiological signature via EEG. The trimodal approach reveals a complementary fingerprint. Neuronal activation within the DMN as assessed with fMRI is positively correlated with the mean standard uptake value of FDG. Electrical source localization of EEG signals shows a significant difference between the dorsal DMN and sensorimotor network in the frequency range of *δ*, *θ*, *α* and *β–1*, but not with *β–2* and *β–3*. In addition to basic neuroscience questions addressing neurovascular-metabolic coupling, this new methodology lays the foundation for individual physiological and pathological fingerprints for a wide research field addressing healthy aging, gender effects, plasticity and different psychiatric and neurological diseases.

## Introduction

The human brain is a very efficient, fast-acting and complex network. A large number of neuronal subnetworks have their own dedicated task and function but continuously share information with each other on a millisecond-by-millisecond basis. One of the most robust neuronal networks is the so-called default mode network (DMN). The canonical DMN comprises precuneus, anterior cingulate cortex (ACC), posterior cingulate cortex (PCC), medial prefrontal cortex (MPfC) and lateral parietal inferior gyri (LPIG)^1–3^. It is thought to characterize basal neural activity^4, 5^ and has been linked to self-referential thought, introspection and integration of cognitive and emotional processing^2^. The DMN shows strong activity during rest, as well as rapid deactivation during externally directed tasks^6^. The DMN is also believed to represent an introspectively oriented mode of the mind, which provides readiness and alertness to changes in the external and internal environment^1^. Functional connectivity within the nodes of the default mode network has a high impact on task performance^7^. However, the energy metabolism of the DMN and its relationship to excitatory and inhibitory neurotransmitters is an open and important question. This is a salient point that needs to be urgently addressed since in disease the hubs of the DMN are most vulnerable^8^ and their energy metabolism might serve as a key biomarker for early disease detection and treatment monitoring. For this diagnostic approach the coupling between neuronal activity, energy consumption, excitatory/inhibitory neurotransmitters and oscillations needs to be investigated. Thus far, such investigations have only been feasible in sequential measurement procedures and are therefore significantly confounded by the fact that data from each modality are recorded under ***different*** physiological conditions. For clinical routine, three different procedures at separate timepoints on compromised patients requiring urgent treatment are unlikely to gain traction in order to bridge the gap between research in basic neuroscience and clinical requirements.

Although fMRI is well-suited for the investigation of functional networks with high spatial resolution, it suffers from limitations in the temporal domain. In order to record temporal patterns of neuronal activity, MRI is not the optimal tool. Neurophysiological measures such as electroencephalography (EEG) measure neuronal activity on a millisecond-by-millisecond basis. A simultaneous MR-EEG approach is technically feasible and unites the high spatial resolution of MRI with the high temporal resolution of EEG. The two main artefacts arising from EEG recordings in an MR-environment, the gradient artefact and the cardioballistic artefact or pulse artefact, can be removed by a combination of different algorithms and ultimately provide EEG data of sufficient quality for further analysis. One of the most salient features of cortical activity is the rhythmic fluctuation of large neuronal assemblies in synchrony^9, 10^. Such oscillations occur over a wide range of frequencies, depending on the behavioural state of the individual^11, 12^. The “basic” activity of the resting state networks consumes up to 80% of the cerebral glucose^13^ and the corresponding glucose consumption can be assessed by FDG PET^14^. According to the work of Riedl and colleagues, increased local activity as measured by FDG-PET is accompanied by higher resting state connectivity as assessed by resting state fMRI^14^. However, Tomasi and colleagues have discussed whether brain regions demonstrating a high degree of functional connectivity are energy efficient and therefore reduce their consumption of glucose considerably as a result of this efficiency^15^. In order to investigate its metabolic profile we studied the default mode network in comparison to the whole brain (this might be confounded by the mix of gray and white matter) and for replication on the network level to another, very stable resting state network, to the sensorimotor network.

By utilising a novel, simultaneous trimodal imaging approach, the fundamental pillars of neuronal activity such as glucose consumption, balance of inhibition/excitation and oscillations can be investigated simultaneously in humans; this is the first such report. In this pilot study we focus our investigation on the following research questions:

- What is the relationship of the BOLD effect - assessed by resting state fMRI - and the metabolic activity measured with FDG-PET in the DMN in comparison to the sensorimotor network?
- Given the high connectivity/central role of the DMN, is its metabolic activity as assessed via FDG-PET higher or lower in comparison to non-DMN regions/another functional network in the brain e.g. the sensorimotor network?
- Do biomarkers of microstructural tissue condition, such as mean diffusivity (MD) - measured by diffusion weighted imaging - differ between the DMN and the sensorimotor network?
- Using single voxel spectroscopy to assess the GABA-to-creatine ratio, how does the most prominent inhibitory neurotransmitter, GABA, in the hubs of the DMN relate to i) the BOLD effect in the DMN and ii) to the metabolic activity in the DMN assessed via FDG-PET?
- Using single voxel MR spectroscopy to assess the glutamate-glutamine to creatine ratio, how does the most prominent excitatory neurotransmitter, glutamate, in the hubs of the DMN relate to i) the BOLD effect in the DMN and ii) to the metabolic activity in the DMN assessed via FDG-PET.
- Is there a significant difference in the source localized EEG data between the DMN and the sensorimotor network? Is there a specific relationship between a certain frequency range of the source localized EEG data with i) mean BOLD signal intensity ii) the metabolic activity assessed via FDG PET in the DMN.

## Methods

### Subjects

In one single session per subject MRI, FDG-PET and EEG data were simultaneously recorded from 11 healthy male volunteers (mean age = 28.6 years, SD = 3.4) in a 3 T hybrid MR-BrainPET system (Siemens, Erlangen, Germany). The study was approved by the Ethics Committee of the Medical Faculty of the RWTH Aachen University and the German administrative body for radiation exposure (Bundesamt für Strahlenschutz). All the methods were performed in accordance with the relevant guidelines and regulations. Prior written, informed consent was obtained from all subjects and the study was conducted according to the Declaration of Helsinki.

### Trimodal Simultaneous Imaging Approach

#### MR data acquisition

The MR data acquisition protocol included the following imaging sequences: magnetization-prepared rapid acquisition gradient-echo (MP-RAGE), ultra-short echo time (UTE), single-voxel spectroscopy, EPI resting state, and diffusion tensor imaging. This protocol was established at the hybrid MR-PET system to optimize simultaneous acquisition efficiency. The detailed parameters for each sequence are listed below.

Anatomical images were acquired for every subject by means of an MP-RAGE sequence (TR = 2250 ms, TE = 3.03 ms, field-of-view (FOV) 256 × 256 × 176 mm^3^, voxel size = 1 × 1 × 1 mm^3^, flip angle = 9°, 176 sagittal slices and a GRAPPA factor of 2 with 70 auto-calibration signal lines).

A UTE sequence (TR = 200 ms, TE_1_ = 0.07 ms, TE_2_ = 2.46 ms, FOV = 320 × 320 × 320 mm^3^, voxel size = 1.67 × 1.67 × 1.67 mm^3^, flip angle = 15°) was used for attenuation correction for the PET data.

Single-voxel MR spectra were consecutively measured using the point resolved spectroscopy (PRESS) sequence (TR = 2500 ms, TE_1_ = 14 ms, TE_2_ = 105 ms, number of averages = 128, 25 × 25 × 25 mm^3^ voxel size, RF pulse centred at 2.4 ppm, 16 step phase cycling) for three areas: PCC, MPfC and precuneus. One extra complete phase cycle for each area was measured without the water suppression RF pulse to record a water peak reference for eddy current correction and absolute metabolite concentration calibration. Before the spectroscopy measurements, the B0 field was shimmed by running FASTESTMAP iteratively to ensure the FWHM of the reference water peak was below 0.05 ppm. The voxel-of-interest was placed sequentially in the PCC, MPfC and precuneus by the same trained operator (JA).

Functional MRI data were acquired using a T_2_*-weighted EPI sequence (TR = 2200 ms, TE = 30 ms, FOV = 200 × 200 × 108 mm^3^, voxel size = 3.125 × 3.125 × 3.0 mm^3^ flip angle = 80°, number of slices: 36). The functional time series consisted of 165 volumes. The subjects were requested to lie down, close their eyes and relax during the six-minute measurement.

Diffusion-weighted data were acquired using a standard double-refocused spin-echo EPI sequence with bipolar gradient pulses (TR = 9100 ms, TE = 87 ms, FOV = 240 × 240 × 136 mm^3^, voxel-size = 1.9 × 1.9 × 1.9 mm^3^, flip angle = 90°, *b*-values = 0, 1000 s mm^−2^, number of slices = 72, number of field gradient directions = 30, number of averages = 4).

#### PET data acquisition

Subjects were instructed to fast overnight and to skip breakfast on the day of imaging. Fasting glucose blood levels were determined prior to imaging. Approximately 200 MBq of FDG was injected via an iv-line at the start of the trimodal imaging experiment, i.e. the volunteer was lying in the hybrid MR-PET scanner at the time of injection. PET data were acquired in list mode for 60 min: the data recorded from 30 to 60 min were averaged and iteratively reconstructed into 153 slices (matrix size 256 × 256, voxel size 1.25 × 1.25 × 1.25 mm^3^ isotropic). The reconstructed images were corrected for attenuation, random and scattered coincidences, dead time and pile up.

#### EEG data acquisition

EEG data were recorded with a Brain Vision Recorder (Brain Products, Gilching, Germany) using a 32-channel MR compatible EEG system including an MR compatible amplifier and a synchronisation box. The EEG cap (BrainCap MR, EasyCap GmbH, Herrsching, Germany) consisted of 31 scalp electrodes distributed according to the 10–20 system and one additional electrode for recording the electrocardiogram (ECG). Data were recorded relative to the Fpz reference and a ground electrode that was located at AFz (10–5 electrode system). Data were sampled at 5000 Hz, with a bandpass of 0.016–250 Hz. Impedances at all recording electrodes were kept below 10 kΩ.

**Figure 1:**
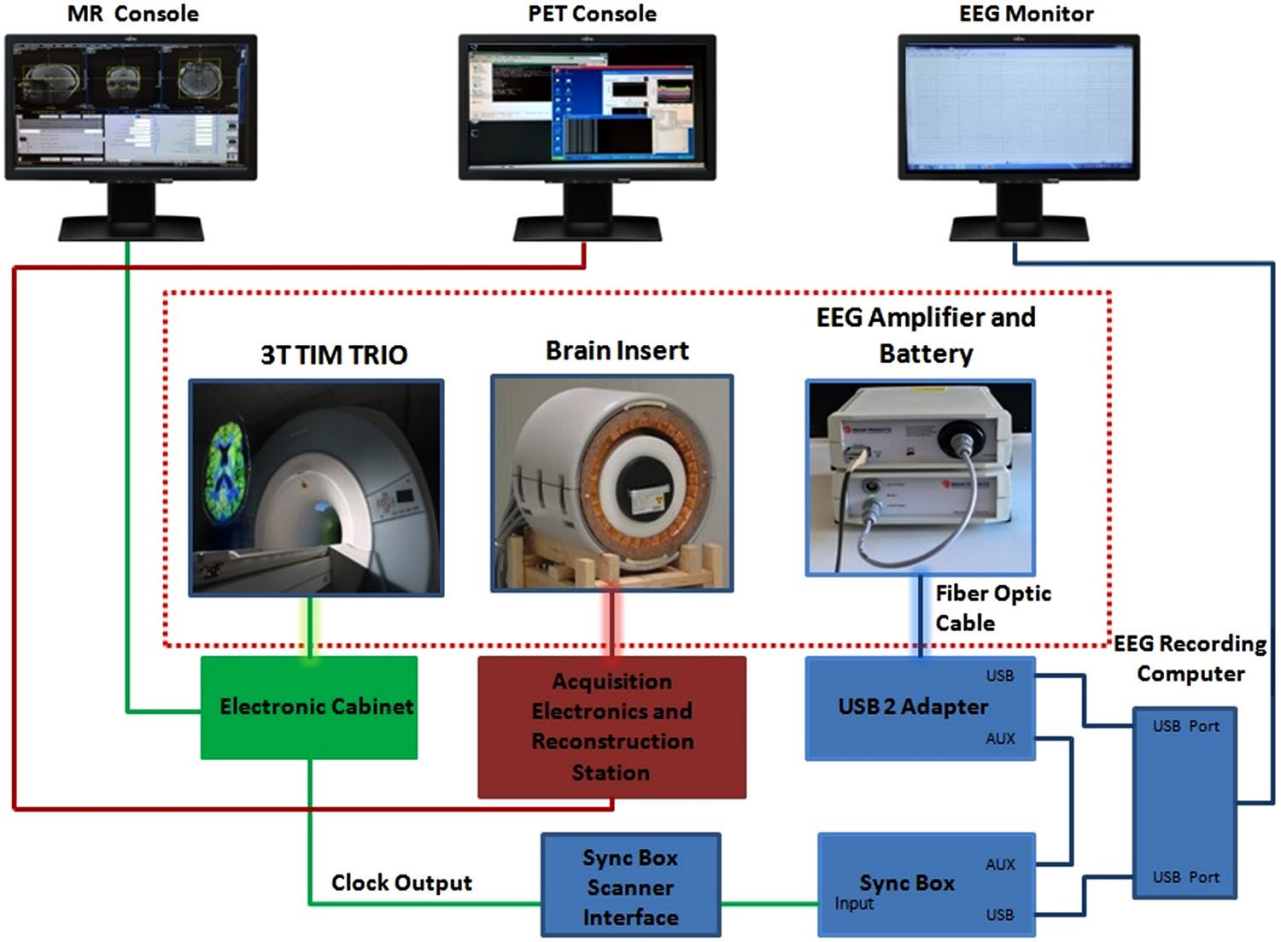
Trimodal set-up. The diagram displays the connections between the various components of the simultaneous MR-PET-EEG setup. The components inside the red dotted rectangular box are inside the RF-shielded MR room.

The simultaneous trimodal data acquisition approach is summarized in Fig. 1.

### Data analysis

#### MRS data analysis

MR spectra were analysed with LCModel version 6.3–0I (http://s-provencher.com/pages/lcmodel.shtml) using a GAMMA simulated basis set^16^ with metabolite chemical shifts and coupling constants taken from the study by Govindaraju *et al*.^17^, provided by the software vendor. The basis set included 16 metabolites. Fitting was performed over the spectral range from 0.2 to 4.0 ppm. The unsuppressed water reference spectrum was used for eddy current correction and water-scaling.

**Figure 2:**
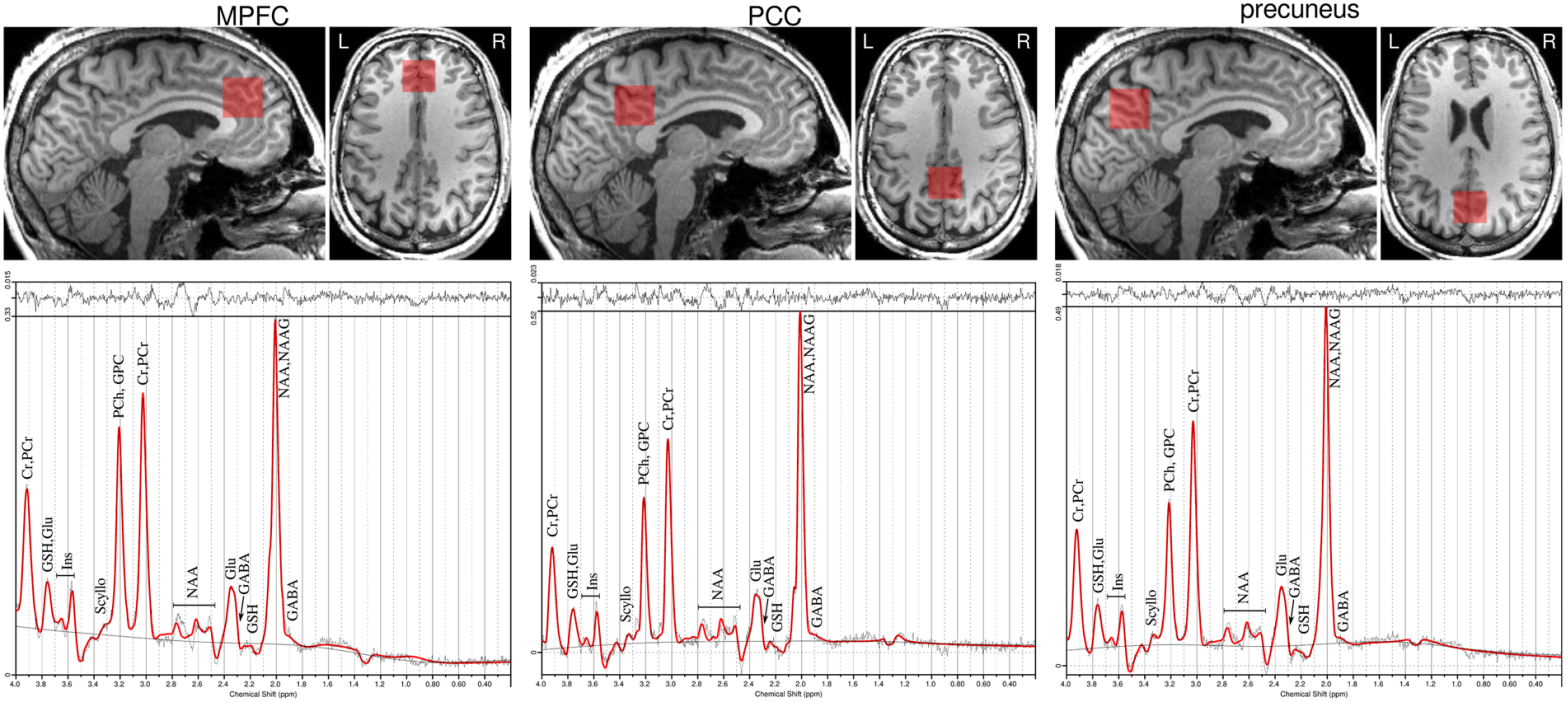
MRS spectra of DMN regions. Positioning of voxels in DMN regions (upper row) and respective MRS spectra (lower row).

The GABA ratio (to Cr + PCr) and glutamate-glutamine ratio (to Cr + PCr) were extracted for each of the three investigated voxels (PCC, MPfC and precuneus) and used for posterior analyses. Metabolite concentrations with the Cramér-Rao lower bound (CRLB) below 20% were considered to be reliably estimated. Example spectra are shown in Fig. 2.

#### FMRI data analysis

Analysis of functional data was carried out using probabilistic independent component analysis^16^ as implemented in MELODIC (Multivariate Exploratory Linear Decomposition into Independent Components) Version 3.10, part of FSL (FMRIB’s software library, www.fmrib.ox.ac.uk/fsl). Individual pre-processing consisted of motion correction using MCFLIRT, brain extraction using BET, spatial smoothing using a Gaussian kernel of full-width at half maximum (FWHM) of 6 mm, and high-pass temporal filtering of 100 s; fMRI volumes were registered to the structural scan of individual and standard space (MNI152) images using FLIRT. Temporal concatenation ICA was performed across all functional datasets from each subject and the number of components to be calculated was set to 20. The DMN was identified by comparison to previously published data^3, 18^ using a correlation coefficient. A mask of the group DMN was created by selecting the voxels with z-scores above 4 and. A grey matter mask created with a group-specific voxel-based morphometry analysis performed in FSL (http://fsl.fmrib.ox.ac.uk/fsl/fslwiki/FSLVBM) and a non-DMN mask was created by extracting the DMN mask from the grey matter mask.

Additionally, in order to compare at the network level, a mask of the dorsal DMN (dDMN) and the sensorimotor network (SMN) was obtained from the 90 fROI atlas^19^ (http://findlab.stanford.edu/functional_ROIs.html).

All these four masks (DMN, non-DMN, dDMN and SMN) were corrected for grey matter using the grey matter mask created. These four masks were used for posterior analyses.

The analysis of the local BOLD signal was carried out as follows: after individual pre-processing and intensity normalisation (grand mean scaling), the mean BOLD signal was extracted individually from the masks previously created.

A Wilcoxon-Mann-Whitney test was performed in the SAS software (version 9.4) to compare a) the mean BOLD signal within the DMN and non-DMN and b) within the dDMN and the SMN. Additionally, bivariate correlation tests were performed using the Spearman rank-order correlation coefficient in the SAS software. Here, the mean BOLD in the DMN mask was tested for correlations with glutamate and GABA ratios the volume-of-interest in the PCC, MLPC and precuneus.

#### Diffusion Weighted Imaging

Pre-processing of the DWI data was performed in FSL (FMRIB’s software library, www.fmrib.ox.ac.uk/fsl). The diffusion data were initially corrected for eddy-current and motion distortions. DTI fitting was performed using FDT (FMRIB’s Diffusion Toolbox) available in FSL. The MD maps were estimated as shown elsewhere^20^ for further analysis. In order to obtain the average MD over the previously created region of interest (ROI) masks (DMN, non-DMN, dDMN and SMN), MD maps as well as the masks were coregistered to the anatomical space of each subject using the toolkit FLIRT. Tissue segmentation was carried out using the MP-RAGE data with the help of the FAST toolkit^21^ available in FSL (http://fsl.fmrib.ox.ac.uk/fsl/fslwiki/). The GM masks were extracted individually for each subject for further analysis taking voxels for which the GM probability was grater than 0.95. Only voxels within both GM mask and ROI masks were used to calculate the average MD for each individual subject.

A Wilcoxon-Mann-Whitney test was performed using the SAS software (version 9.4) to compare the mean MD in DMN and non-DMN as well as dDMN and SMN.Additionally, bivariate correlation tests were performed using the Spearman rank-order correlation coefficient using the SAS software. Here, the mean MD within the DMN mask was tested for correlations with glutamate and GABA ratios in the PCC, MLPC and precuneus

#### PET data analysis

Evaluation of the PET data was based on the reconstructed images showing the cerebral uptake of FDG averaged from 30 to 60 min after injection. The PET images were converted to standard uptake value (SUV) maps accounting for the injected dose and body weight of every subject. The mean SUV was extracted from a) the DMN and non-DMN masks and b) in addition, at the network level, from the dDMN and SMN individually. In order to carry out the normalisation, the mean SUV in the whole brain grey matter mask was also calculated. The grey matter mask was created with a group-specific voxel-based morphometry analysis performed in FSL software. The extracted mean SUVs were normalised to whole-brain grey matter SUV using formula (mean SUV in ROI mask/mean SUV in whole-brain grey matter mask) and exported for further statistical analysis. A Wilcoxon-Mann-Whitney exact test was performed using the SAS software (version 9.4) to compare the mean SUV in the DMN and the non-DMN as well as in the dDMN and the SMN.

Additionally, bivariate correlation tests were performed using the Spearman rank-order correlation coefficient using the SAS software. Here, the mean SUV of FDG in the DMN was tested for correlations with glutamate and GABA ratios in the PCC, MLPC and precuneus.

#### EEG data analysis

EEG data were processed using Brain Vision Analyzer (Version 2.0. Brain Products, Munich, Germany). Gradient artefact correction was performed using the method proposed by Allen *et al*. and included in Brain Vision Analyzer^22^. Data were down-sampled to 250 Hz and a low-pass filter with a cut-off frequency of 40 Hz was applied. Detection of the heartbeat event in the ECG signal was performed in Brain Vision Analyzer. Data were exported to EEGLAB^23^ where optimal basis set (OBS)^24^ analysis was performed using the toolbox available in this software; the number of principal components to use was set to 3. The markers of heartbeat events were used for pulse artefact correction. The data were later exported back to Brain Vision Analyzer for further analysis. Re-referencing of the data was carried out including all EEG channels as a new reference. For further de-noising of the data, independent component analysis was applied to the data using the extended Infomax algorithm. The entire-length EEG data were used for the calculation of the independent components. The selection of artefactual components was based on the spectral information provided by wavelet analysis of each IC according to Maggioni *et al*. ^25^ where the ICs having a peak time locked to the R peak between the delta and alpha band were selected and removed. For calculation of the wavelets, the following parameters were used: complex morlet family of wavelets (minimal frequency = 0.5 Hz, maximal frequency = 30 Hz, frequency steps = 300, morlet parameter c = 10, linear steps).

The de-noised data were later exported to the LORETA-KEY software (http://www.uzh.ch/keyinst/loreta.htm), where cross-spectra of the EEG signals were computed for each subject. A 3D cortical distribution of the neuro-electrical generators was computed using eLORETA. The frequency bands were *δ* (1.5–6 Hz), *θ* (6.5–8 Hz), *α* (8.5–12 Hz), *β–1* (12.5–18 Hz), *β–2* (18.5–21 Hz) and *β–3* (21.5–30 Hz). For solving the forward problem, eLORETA used a three-shell spherical head model registered to a standardized stereotactic space available as digitized MRI data from the Brain Imaging Centre (Montreal Neurological Institute, MNI305). Registration between spherical and realistic head geometry was performed using the EEG electrode coordinates as implemented in the LORETA-KEY software^26^. Individual maps for each subject for each frequency range were Z-score normalised (relative to the global mean and standard deviation), exported, and the mean activity of the electrical generators within the dDMN mask was extracted, as well as within the SMN mask. Wilcoxon signed-rank tests were performed in the SAS software to compare the mean electrical sources of each frequency inside the dDMN and SMN. The Wilcoxon signed-rank test is appropriate because the assumption of a Gaussian distribution was not valid for the electrical sources of the EEG frequencies.

Additionally, bivariate correlation tests were performed using Spearman’s rank-order correlation in SAS. Here the electrical sources of each frequency were tested for correlations with glutamate and GABA ratios in the PCC, MLPC and precuneus.

### Data Availability

The datasets generated and analysed during the current study are not publicly available due to restrictions in ethical approval granted by the Ethics Committee of the RWHT Aachen University to the Forschungszentrum Juelich but are available from the corresponding author on reasonable request.

## Results

### MRS data

The glutamate ratio in the precuneus (GlutPREC), the GABA ratio in the precuneus (GABAPREC), the glutamate ratio in the PCC (GlutPCC) and the GABA ratio in the PCC (GABAPCC) were successfully measured using single voxel MRS. GlutPREC (mean = 0.903, SD = 0.031) exhibited a normal distribution according to a Shapiro-Wilk test (significance value = 0.464); GABAPREC (mean = 0.165, SD = 0.018) exhibited a normal distribution according to a Shapiro-Wilk test (significance value = 0.265); GlutPCC (mean = 0.898, SD = 0.079) exhibited a normal distribution according to a Shapiro-Wilk test (significance value = 0.134); and GABAPCC (mean = 0.151, SD = 0.031) exhibited a normal distribution according to a Shapiro-Wilk test (significance value = 0.835). Glutamate and GABA ratios in the MPfC could not be reliably estimated in two volunteers due to poor magnetic field shimming. As such, these parameters were not included in the analyses and results.

### FMRI data

**Figure 3:**
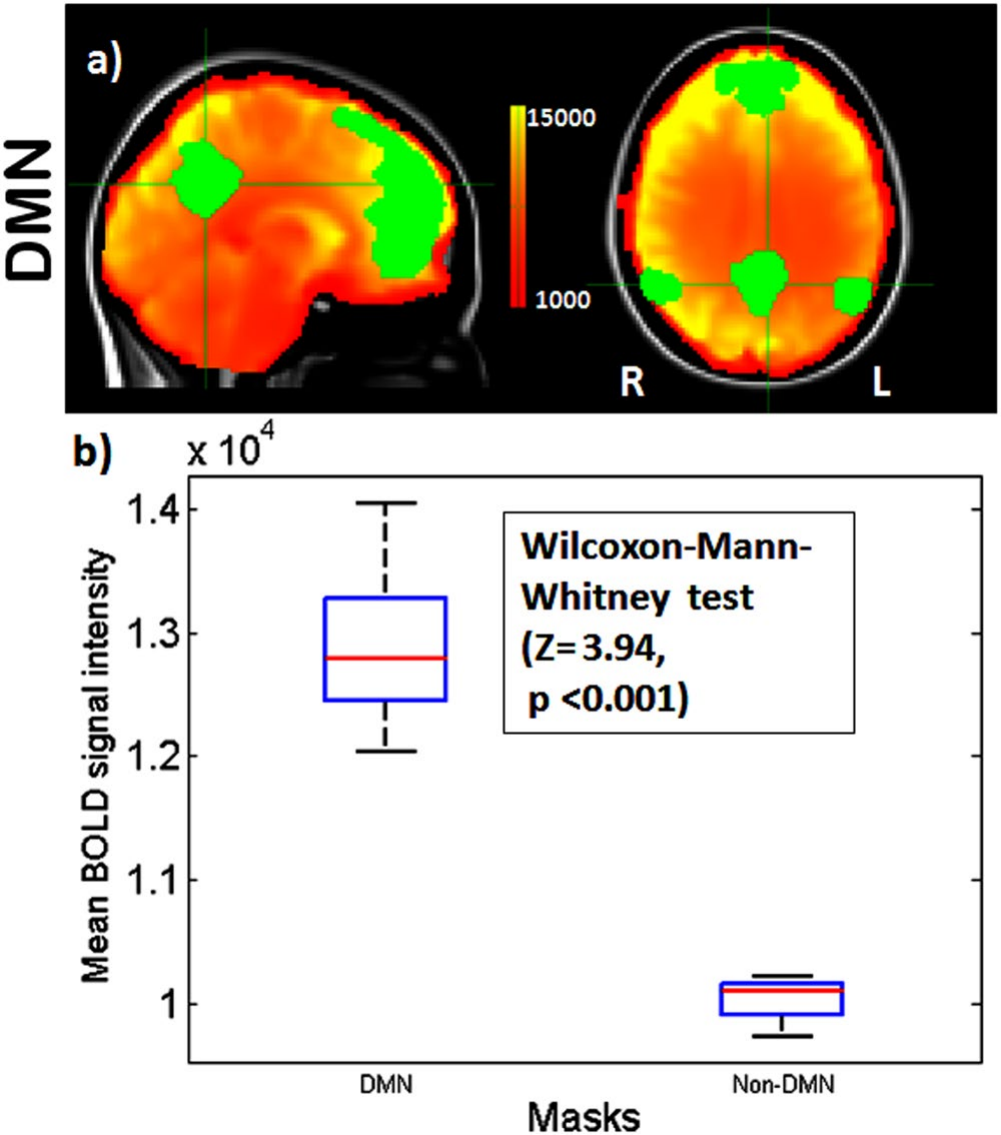
fMRI BOLD signal within the DMN and Non-DMN. (**a**) Resting state mean BOLD image of an exemplary subject with DMN mask identified by ICA overlaid on MNI 152 atlas (upper row). (**b**) The box plot shows the mean BOLD signal in within the DMN and non-DMN mask. During rest the BOLD signal is significantly higher in the DMN than outside.

Decomposition of the data by means of ICA identified the DMN in accordance with the study by Smith *et al*.^18^. The correlation coefficient for comparison between the DMN in our sample and in the sample by Smith *et al*.^18^ was *r* = 0.47 (p < 0.00001). The Wilcoxon-Mann-Whitney test showed that the mean BOLD signal in the DMN mask was higher than outside the DMN mask (Z = 3.94, two sided p < 0.001) (Fig. 3).

**Figure 4:**
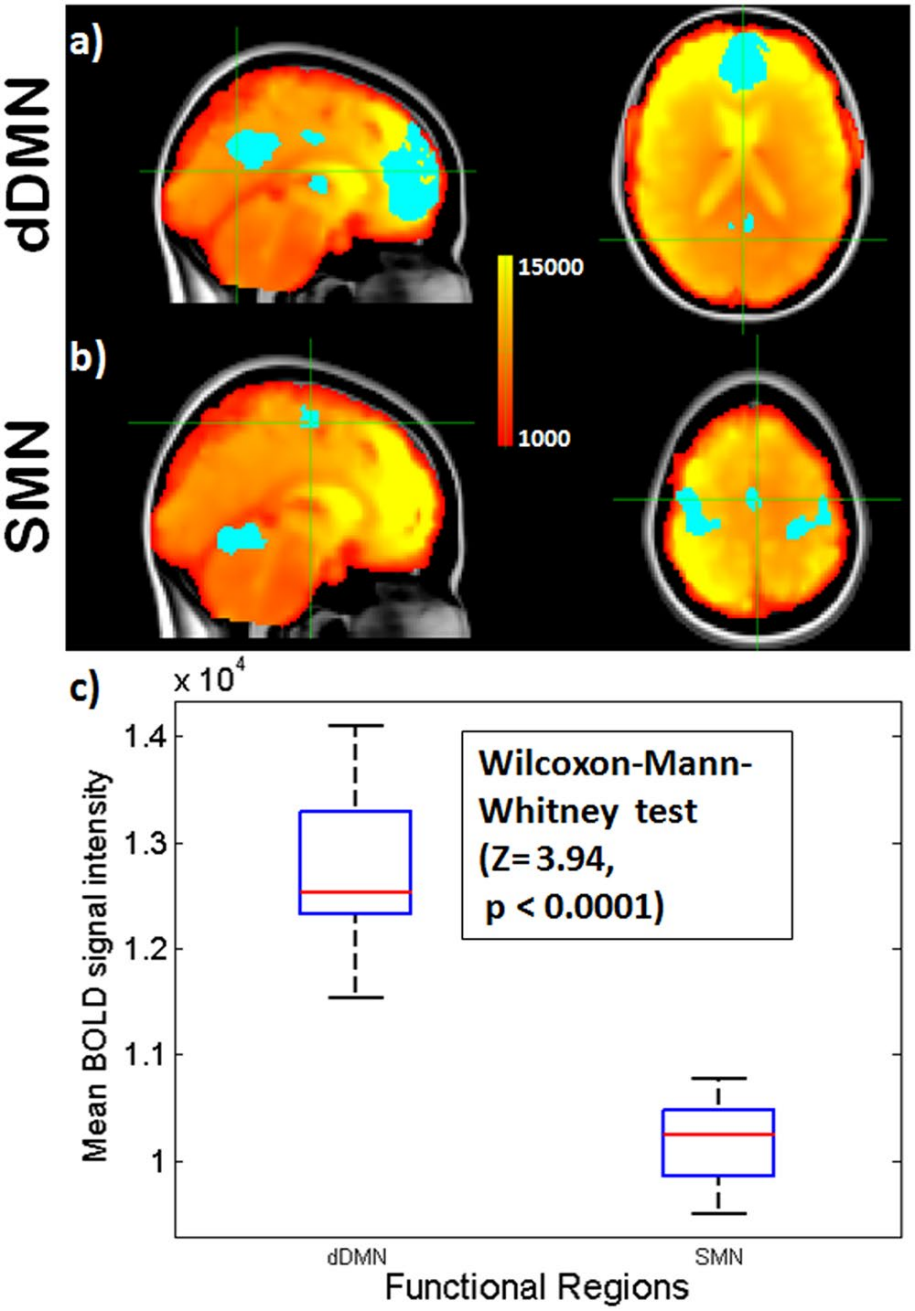
fMRI BOLD signal within the dDMN and SMN. Resting state mean BOLD image of an exemplary subject with masks of the functional regions of the (**a**) dDMN and (**b**) SMN overlaid on MNI 152 atlas (upper two rows). (**c**) The boxplot shows the mean BOLD signal in within the dDMN and SMN for 11 subjects. During rest the BOLD signal is higher in the dDMN than in the SMN.

This finding was replicated at the network level: the Wilcoxon-Mann-Whitney test showed that the mean BOLD signal in the DMN mask was higher than in the SMN mask (Z = 3.94, two sided p < 0.0001) (Fig. 4).

The Spearman rank-order correlation did not show any statistically significant correlation between GlutPREC and mean BOLD signal in the DMN (r_s_ = 0.26, n = 11, p = 0.43). This was also the case for GABAPREC (r_s_ = −0.26, n = 11, p = 0.44), GlutPCC (r_s_ = 0.30, n = 11, p = 0.35) and GABAPCC (r_s_ = 0.01, n = 11, p = 0.98).

### Diffusion weighted imaging data

**Figure 5:**
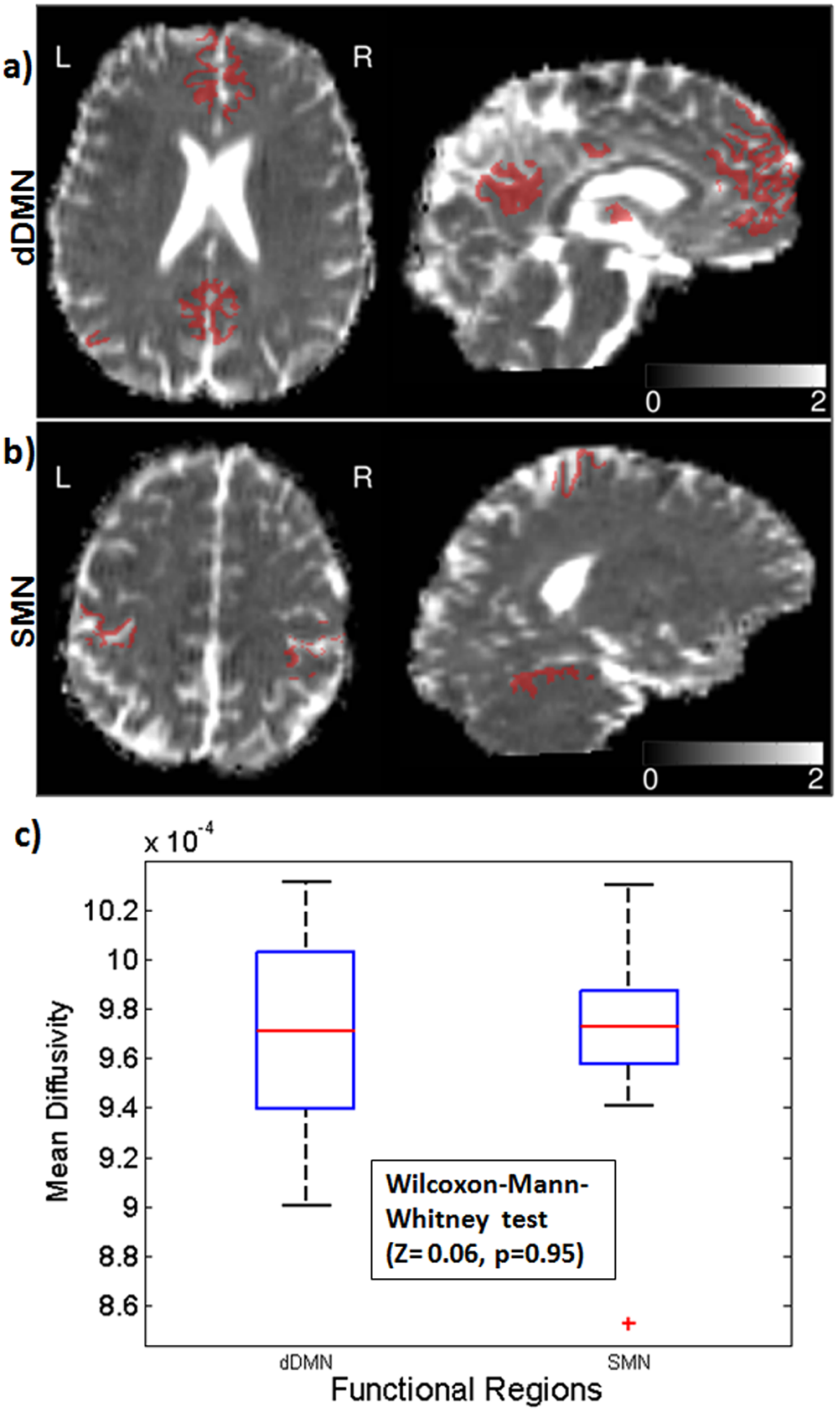
Mean Diffusivity within the dDMN and SMN. (**a**) dDMN and (**b**) SMN masks overlaid on a representative MD map. (**c**) The boxplot shows the mean MD in the dDMN and SMN mask for 11 subjects.

The Wilcoxon-Mann-Whitney test did not show any significant difference between the mean MD in the DMN and in non DMN (Z = 1.44, two sided p = 0.15) as well as in dDMN mask and in SMN mask (Z = 0.06, two sided p = 0.95) (Fig. 5).

The Spearman rank-order correlation did not show any statistically significant correlation between GlutPREC and mean MD in the DMN (r_s_ = −0.19, n = 11, p = 0.57). This was also the case for GABAPREC (r_s_ = −0.17, n = 11, p = 0.61), GlutPCC (r_s_ = 0.004, n = 11, p = 0.99) and GABAPCC (r_s_ = −0.19, n = 11, p = 0.57). This was also the case for mean BOLD intensity (r_s_ = 0.32, n = 11, p = 0.34) and normalised mean SUV of FDG (r_s_ = 0.28, n = 11, p = 0.40).

### PET data

**Figure 6:**
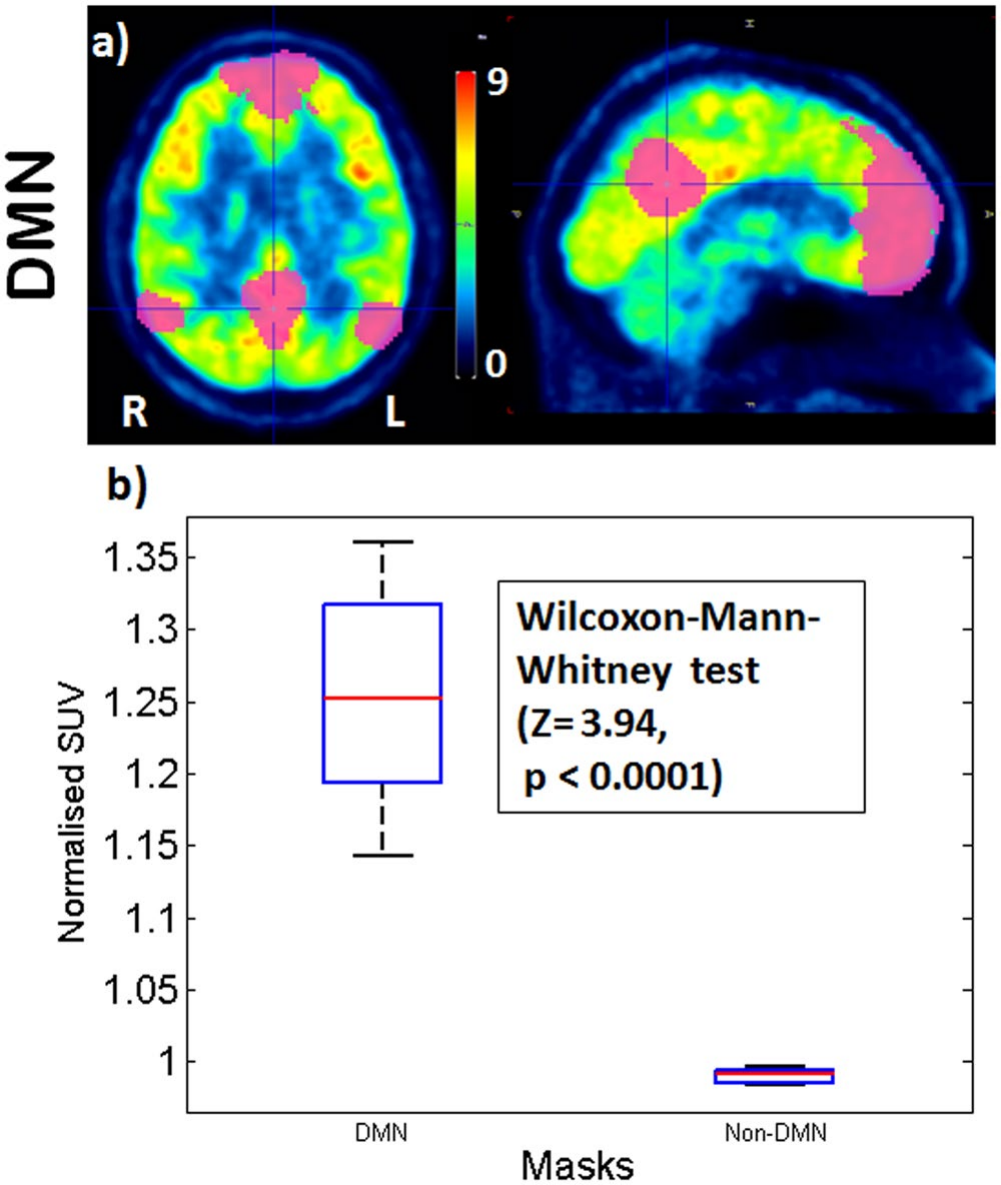
SUV of FDG within the DMN mask and Non-DMN. (**a**) Resting state FDG PET SUV image of an exemplary subject with DMN mask identified by ICA (upper row). (**b**) The box plot shows the normalised SUV of FDG within the DMN mask and the non-DMN mask for 11 subjects.

The Wilcoxon-Mann-Whitney test showed that the SUV in the DMN mask was higher than outside the DMN mask (Z = 3.94, p < 0.0001) (Fig. 6).

**Figure 7:**
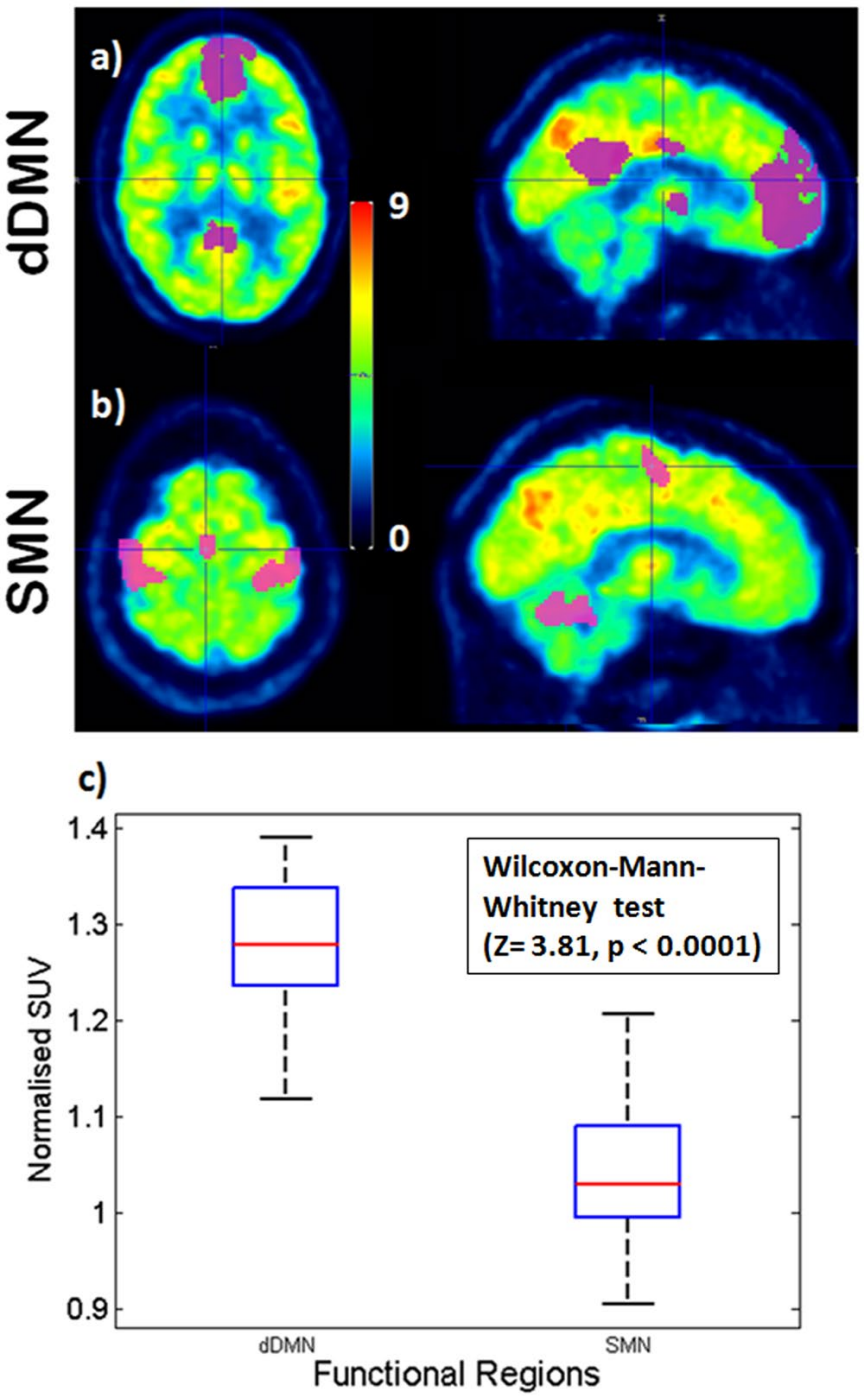
FDG SUV in the dDMN and SMN. Resting state FDG PET SUV image of an exemplary subject with (**a**) dDMN and (**b**) SMN masks (upper two rows). (**c**) The box plot shows the normalised SUV in dDMN mask and SMN mask for 11 subjects.

In analogy, the Wilcoxon-Mann-Whitney test showed a significant difference between the SUV in the dDMN mask and in SMN mask (Z = 3.81, two sided p < 0.0001) (Fig. 7).

**Figure 8:**
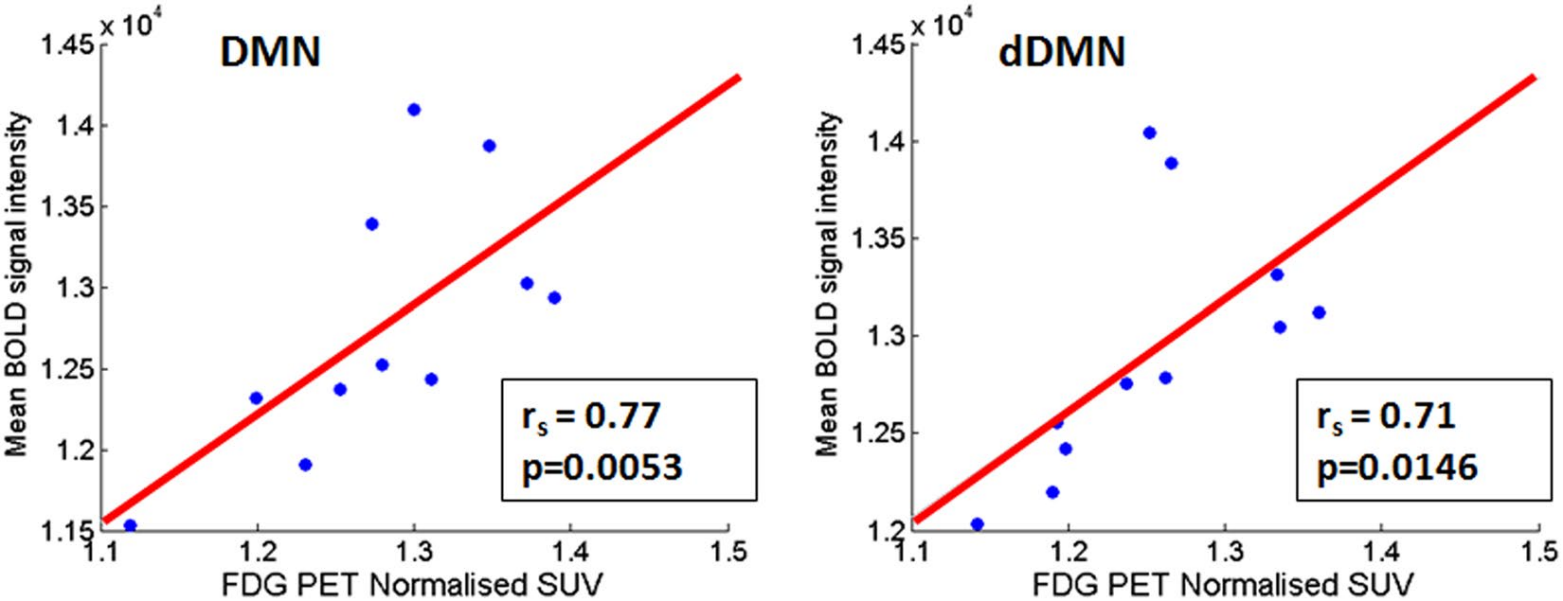
Correlation plot between normalised SUV uptake of FDG PET and BOLD signal intensity in DMN (right side) and dDMN (left side).

The Spearman rank-order correlation shows a statistically significant positive correlation between BOLD signal intensity and normalised SUV of FDG in DMN (r_s_ = 0.77, n = 11, p = 0.0053) and dDMN (r_s_ = 0.71, n = 11, p = 0.0146) (Fig. 8).

The Spearman rank-order correlation did not show any statistically significant positive correlation between GlutPREC and the normalised SUV in the DMN (r_s_ = 0.09, n = 11, p = 0.79). This was also the case for GABAPREC (r_s_ = −0.17, n = 11, p = 0.61), GlutPCC (r_s_ = 0.29, n = 11, p = 0.39) and GABAPCC (r_s_ = −0.11, n = 11, p = 0.74).

### EEG data

**Figure 9:**
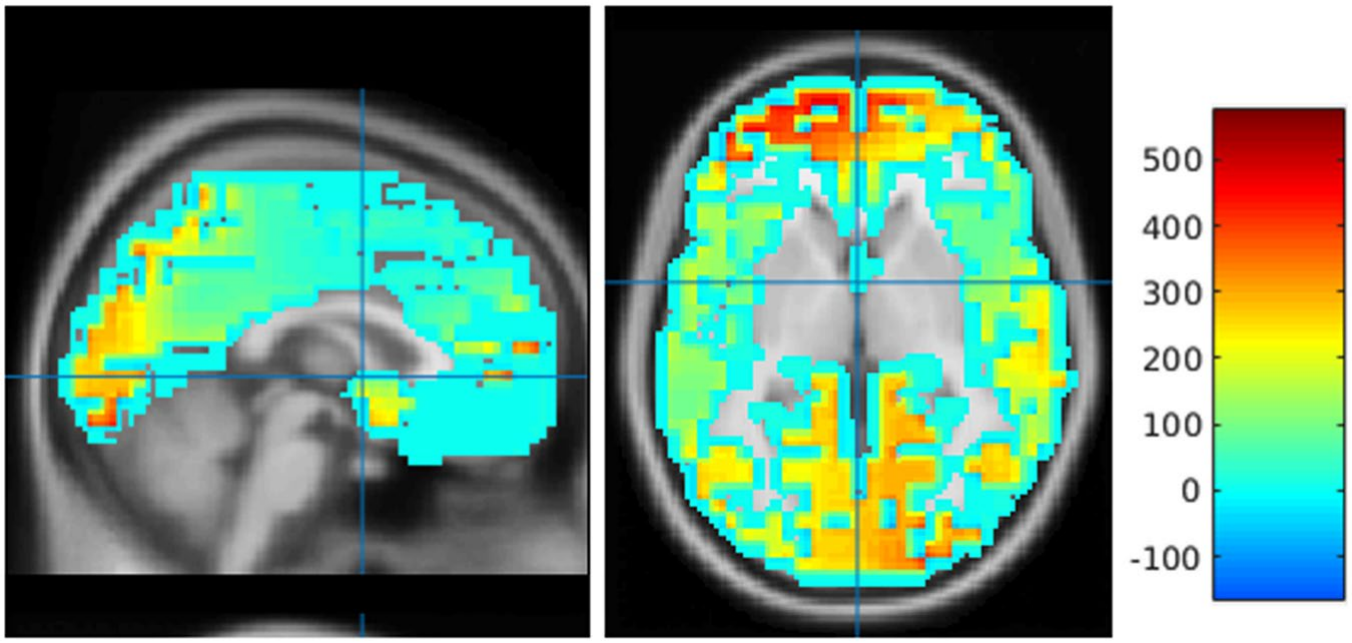
A 3D cortical distribution of the neuro-electrical generators of EEG alpha frequency band of a representative subject. The neuro-electrical generators computed using eLORETA is overlaid on a sagittal and axial slice of the MNI template.

A 3D cortical distribution of the neuro-electrical generators was successfully computed from denoised EEG data for frequency bands *δ*, *θ*, *α* (Fig. 9), *β–1*, *β–2* and *β–3* using eLORETA.

The Wilcoxon-Mann-Whitney test showed a significant difference in the electrical sources between the DMN and SMN in *δ* (Z = 3.35, two sided p = 0.0003), *θ* (Z = 3.22, two sided p = 0.0006), *α* (Z = 3.09, two sided p = 0.001) *and β–1* (Z = 2.76, two sided p =  < 0.0041). This was not the case for *β–2* (Z = 0.2, two sided p = 0.85) and *β–3* (Z = 0.98, two sided p = 0.33).

Spearman’s rank-order correlation tests did not show any significant correlation between GlutPREC and electrical sources of *δ*, *θ*, *α*, *β–1*, *β–2 and β–3 band EEG frequencies*. This was also the case for GABAPREC, GlutPCC, GABAPCC, mean BOLD intensity and normalised mean SUV of FDG.

## Discussion

An approach for simultaneous trimodal imaging was successfully developed and implemented in humans for the first time. This novel methodology revealed significantly higher metabolic activity in the default mode network of the brain in comparison to structures outside the DMN. The higher metabolic activity is coupled in our sample with higher BOLD amplitudes. This finding is also replicated in the direct comparison between dDMN and SMN at the network level. However, there was no significant link between a given EEG frequency band and the metabolic activity as assessed for the comparison between the DMN and non-DMN or the dDMN and SMN. However, at the network level there was a significant difference between the dDMN and SMN for the electrical sources of frequencies in the range of *δ*, *θ*, *α* and *β–1*.

### Relationship/coupling of metabolic behaviour and neuronal activity in the default mode network of the human brain

The default mode network has a salient function in the functional network architecture of the brain. On one hand it, has been shown in prior studies that regions of elevated topological importance in resting state networks also have particularly high metabolic demands^13^. But on the other hand, important hubs such as the default mode network might operate in a specialized, energy-efficient mode^15^. Based on the trimodal imaging approach, our data in healthy male volunteers show that the higher neuronal activation - as indicated indirectly via the higher BOLD signal intensity in the dDMN structures - is coupled to a higher normalised SUV of FDG. These results are in agreement with a bimodal MR-PET study by Riedl and colleagues^14^ showing that metabolic activity as assessed via FDG PET is coupled with the resting state BOLD.

### Relationship between microstructure and function in the DNM and in non-DMN regions

Honey and colleagues have reviewed different network modelling studies^27^ and have addressed the question of whether “structure (could) predict function in the human brain”. They concluded that structure and function are closely related. Furthermore they emphasize that the relationship is particularly robust for functional networks exhibiting low frequencies such as those from resting state fMRI data and when the resting state fMRI data are sampled over a period of a few minutes.

In our data the biomarker for microstructural properties MD did not show a significant difference between the dDMN and SMN while the functional networks did exhibit a significant different metabolic profile as indicated by the FDG PET data. In other words also the two different resting state networks have a comparable structure its metabolic demands differ as indicated by the higher SUV in the DMN^27–31^. In most of the diffusion MRI investigations, the observed changes in MD are associated with changes in the tissue microstructure, since water molecules serve as a probe of the the tissue geometrical microenvironment. Significant correlations of the absolute concentration of Glutamate with MD in the PCC have previously been observed during rest in normal volunteers^32^ as well as in long-term Zen meditators^33^. In Arrubla *et al*.^32^, it was hypothesised that this correlation can *a priori* involve both microstructural and (transient) physiological changes. In particular, the observed negative correlation of MD with the glutamate concentration was interpreted as a subtle cell swelling arising as a consequence of neuronal activation driven by the increase in the glutamate concentration, given that the PCC shows strong activation during rest. In this study, the lack of a negative correlation of MD with glutamate could be due to the fact that here MD was observed in the whole DMN network, i.e., not only the PCC as in Arrubla *et al*.^32^. Moreover, the small subject sample investigated here can also play a role in the observed results. Therefore, these observations needs further investigation in the framework of dedicated studies.

### Relationship between GABA/Glutamate as main mutual drivers of the inhibition/excitation balance in the human brain and measures of metabolic and neuronal activity

The amplitude of neuronal activity in the human central nervous system is determined by the balance of excitation and inhibition by synaptic inputs from the major inhibitory neurotransmitter GABA and the major excitatory neurotransmitter glutamate^34^. This balance between neuronal inhibition and excitation is governed, to a large degree, via a negative feedback mechanism that results in neuronal inhibition. Here, the key mechanisms in healthy brain tissue are inhibitory GABAergic interneurons^34^ and glutamate release steered by presynaptic metabotropic glutamate receptors^35^.

### Relationship of GABA to BOLD amplitude and SUV of FDG PET

Focusing on GABA and BOLD, Donahue and colleagues^36^ summarized the current status quo: “It is unclear precisely what role GABA may play on the hemodynamic constituents of the BOLD signal. One hypothesis is that neuronal firing rates and in turn energy utilization will be lower in regions of high GABA. Alternatively, a positive correlation could be possible since inhibitory firing could result in increased energy consumption, or, higher baseline inhibition could result in a higher level of hemodynamic and metabolic response necessary to overcome the baseline inhibition.” In our trimodal data no correlations between GABA and mean BOLD signal intensity within the DMN network were found.

One prior experiment showed a negative correlation between GABA and intrinsic functional connectivity in the posterior medial frontal cortex as assessed from resting state fMRI^37^. In a prior bimodal study (simultaneous MRS-EEG) in healthy male volunteers we also saw no correlation of GABA with the BOLD amplitude as measured in the PCC in the DMN but we did see a correlation with the BOLD response of the putamen^38^. Further studies exploiting larger sample sizes are needed to determine whether there is really no correlation between the functional connectivity within the DMN or whether there is a small effect of GABA e.g. via the GABA-ergic interneurons and our negative result is a false negative one due to the small sample size of this explorative pilot study.

In our simultaneously acquired data there is no significant relationship between GABA and SUV of FDG. This is in line with *in vitro* studies by Chatton and colleagues^39^. They reported that GABA uptake into astrocytes is not associated with significant metabolic cost, which suggests that GABA may not couple inhibitory neuronal activity with glucose utilization and thus not contribute directly to brain imaging signals associated with transient glycolytic processing of glucose^40^.

### Relationship of Glutamate to BOLD amplitude and SUV of FDG PET

Regional metabolic activity as determined by ^18^FDG-PET largly reflects the influence of glial uptake of glucose in response to neuronal glutamate release^35, 40^. A close link between glutamate and mean glucose consumption has been demonstrated in anaesthetized cats^41^. This finding is in line with the observation that the major part of brain energy consumption is dedicated to excitatory activity^41, 42^. However, in our human pilot sample, this relationship was not replicated. In our small cohort of young healthy volunteers no correlation was detected. This would point in the direction that under physiologic conditions during rest the inhbition/exhibition balance is kept^42^. Larger human cohorts are needed in the future to investigate this mutual link with more statistical power.

### Oscillatory activity in relation to hemodynamic activity

Local and large-scale networks communicate via different frequency ranges. As EEG provides high temporal resolution it allows for the separation and extraction of slow and fast oscillatory neuronal activit^10^. GABAergic interneurons play a key role in the level of inhibitory tonus. GABAergic interneurons are critical elementary units, maintaining important spatial and temporal functionality in cortical circuits. Without inhibitory interneurons, networks of excitatory neurons would converge on a state of increasing or saturated excitation, incapable of processing information in a meaningful ways^34, 43^.

Dumitriu and colleagues observed an association between different subtypes of GABAergic interneurons and frequency bands. It has been observed for example that parvalbumin interneurons are fast-acting on the soma and “entrench gamma bursts to optimize coincidence detection”^34, 44^. In contrast, other interneurons (somatostatin and neuropeptide Y) exhibit slower kinetics and acting at apical dendrites triggering oscillatory activity in the form of theta oscillation^34, 44^.

Prior investigations showed a significant correspondence between BOLD functional connectivity and synchronized neuronal activity in the gamma frequency band (>30-Hz)^25^. Work in animals (anaesthetised cats subjected to visual stimulation) demonstrated a correlation between the strength of hemodynamic responses and the oscillatory power of several frequency bands^45^. The strongest link was established between oscillatory power in the upper part (>60 Hz) of the gamma frequency band and the strength of the hemodynamic response^13, 45^. Another study recorded simultaneously resting state fMRI and intracortical electrophysiological data in the visual cortex of anaesthetised monkeys^46^. fMRI data correlated strongest in the gamma band with some fluctuation in neuronal spiking activity as recorded by local field potentials (LFP). Synchronous activity of GABAergic interneurons is supposed to be a key mechanism in the generation of gamma oscillations in local circuits^13^.

In our human *in vivo* data with surface electrophysiological recordings no correlation between the power of any frequency band and the amplitude of the BOLD effect in the DMN was detectable. However, we detected a significant difference when comparing the electrical sources for delta, theta, alpha and beta 1 between the DMN and SMN. These frequencies play an important role in the long-range synchronization for the effective coupling between more remote brain regions^47^. However, no frequency band in the DMN correlated to the GABA or glutamate measures.

### Oscillatory activity in relation to metabolic activity

Results from human clinical studies show a high metabolic demand associated with gamma oscillations^48^. In patients suffering from non-lesional focal epilepsy cortical glucose metabolic patterns showed a strong correlation with the gamma spectral amplitude^13, 48^. No correlations between glucose consumption rate and subdural EEG measures in the delta, beta, theta and alpha frequency band were described. Again these data were recorded invasively and are not replicated in our human data in healthy volunteers.

This explorative pilot study in humans has some limitations. The sample size is small given the number of parameters assessed. However, it demonstrates the successful implementation of the trimodal simultaneous approach in general. Findings such as the strong correlation between BOLD and SUV in the DMN are fully in line with findings in the literature^49^ but nevertheless needed to be replicated.

The EEG approach with surface electrodes suffers from the implicit methodological confound of the inverse problem. Our approach via eLORETA can only provide estimated sources at low spatial resolution.

The true advantages of the simultaneous approach will come to the fore in studies employing pharmacological challenges, where two (or even three) different time points in non-simultaneous measurements suffer always from confounding factors. Another domain is the application of trimodal imaging in patients in clinical neuroscience who are unable to undergo all three different modalities at three different time points and who need quick pharmacological intervention with a minimum waiting time resulting from diagnostic procedures (e.g. therapy resistant epilepsy, prediction of therapeutic response to medication such as serotonin reuptake inhibitors or atypical neuroleptics). The trimodal approach holds great promise due to the richness and complexity of the data acquired in different dimensions and time scales; this has enormous potential for the development of individual biomarkers in form of the multimodal fingerprint for individual prognosis and individualized treatment planning e.g. in schizophrenia, depression and in the mid-term, potentially, dementia.
